# How Do Career Development Courses Help Chinese Undergraduate Students Achieve Healthy and Quality Career Development?

**DOI:** 10.3390/ijerph192315620

**Published:** 2022-11-24

**Authors:** Teng Zhao, Jingchao Wu

**Affiliations:** Zhejiang Academy of Higher Education, Hangzhou Dianzi University, Hangzhou 310018, China

**Keywords:** career development courses, career awareness, job search self-efficacy, career planning ability, healthy and quality career

## Abstract

Postsecondary institutions worldwide generally provide career development courses or similar courses to better prepare undergraduate students for healthy and quality future careers. Understanding whether these career development courses positively affect students’ career-related outcomes is crucial. Utilizing survey data collected from a large research university located in the eastern part of China, we found that students who have taken at least one career course exhibited career awareness and career planning abilities that were 0.096 and 0.147 units higher, respectively, than those of students who have not taken career courses, with other variables held constant. More specifically, an additional career course was statistically significantly associated with a 0.099, 0.084, and 0.175 unit increase in students’ career awareness, job search self-efficacy, and career planning ability, respectively. A student’s college major and annual family income seemed to be good predictors for a student’s career awareness, job search self-efficacy, and career-planning ability. Furthermore, the more career courses that a student took, the higher the career awareness, job search self-efficacy, and career planning ability that the student had. With these findings in mind, our study recommends postsecondary stakeholders to leverage such courses to help students better prepare for a healthy and quality career development.

## 1. Introduction

Currently, college students face a complex and changing world influenced by globalization, multiculturalism, changing organizational structures, and flexible employment patterns [1]. Combined with the influence of COVID-19, the workforce is more mobile and fragile than ever before, which may harm students’ career-related mental health. There is a strong need for college students to gain transferable knowledge and skills to be better prepared for unexpected career changes [2]. Therefore, postsecondary institutions are also pressured to help students prepare for healthy and quality careers and achieve success [2,3], especially by helping those who are undecided about their future paths. One approach is to offer career development courses designed to meet the needs of students who are in the deciding phase of selecting a career or major [4].

Career development courses have existed in postsecondary institutions for a long time [5]. Colleges and universities across the United States have offered formal career development courses for over 90 years [6]. The Australian government published an *Australian Blueprint for Career Development*, which showed a framework for university lecturers teaching career development courses [7]. The Ministry of Education of New Zealand advocated a whole-school approach to career guidance and education in 2009, and many universities and polytechnics provide students with career development courses [8].

With the evolution of the career development course, scholars are dedicated to clarifying whether this kind of course is helpful. Researchers in various countries have tested the efficiency of career development courses in different majors, such as sociology, psychology, chemistry, and medicine [3,9,10,11]. Additionally, research on this topic has been published since 1970. Sufficient evidence could support career development courses positively impacting students’ cognitive function and behavior outcomes [5,12]. Firstly, the existing research has found that career development courses can inspire students’ career thoughts [13], helping students to determine the type of job that they would like to have [3]. Secondly, past literature has noted that career development courses can encourage students to learn more about their intended careers [10,14] and work life [1], furthering their skills in order to help them transfer to a professional staff beyond just having related professional knowledge [11]. Thirdly, some literature has pointed out that career development courses positively affect students’ transitions and future planning [2,4,15]. These previous studies connect career development courses with career awareness, job search self-efficacy, and career planning ability.

In 2007, the General Office of the State Council of China stated that career development courses should be embedded in tertiary education. In the same year, the Ministry of Education (MOE) also indicated that all colleges and universities should offer career courses [16]. According to a national survey of 106 universities in China, 100% reported offering at least one career development course [17]. For the present study, a large research university in eastern China mandatorily requires all undergraduate students to enroll in a series of four compulsory career development courses named “Career Development and Employment Guidance” within four semesters, starting from sophomore to junior year. They are all multimedia lecture courses, with different essential sections: self-exploration, career planning, job searching, and practical skills. These compulsory career development courses are only offered once a week in the first eight weeks of the semester. In addition to these courses, students are also allowed to enroll in other optional career development courses such as “Psychological Types and Career Development” depending on their needs, providing a unique opportunity to investigate how career development courses influence undergraduate students.

This study aims to understand the relationships between career development courses and undergraduate students’ career-related outcomes, such as career awareness, job search self-efficacy, and career planning ability. These career-related concepts were originally from Western countries and have largely been examined by Western samples. To the authors’ knowledge, there is insufficient literature on whether career development courses jointly contribute to students’ career awareness, job search self-efficacy, and career planning ability from a non-Western perspective. Though Chinese governments mandate universities and colleges to offer career development courses, the effects of these courses are insufficiently examined. Thus, our study offers both research contributions—to expand our lens into how career courses contribute to students’ career-related outcomes—and practical contributions, informing postsecondary stakeholders of the effects of career courses when designing undergraduate curriculums, and thereby better preparing students for healthy and quality careers. Leveraging the data collected by a self-administrated survey titled “The Effectiveness of College Students’ Career Development Courses” (ECSCDC), we mainly focus on the following research questions: (1) What is the relationship between career development courses and students’ career awareness?; (2) What is the relationship between career development courses and students’ job search self-efficacy?; and (3) What is the relationship between career development courses and students’ career planning ability?

## 2. Theories and Hypotheses Development

### 2.1. Career Awareness

Career awareness is the starting point of the career development process. If students have career awareness, will they be able to think about their career plans and explore different careers? Career awareness helps to lay the foundation for students’ successful career exploration and planning [18]. It also contributes to students’ career decision-making self-efficacy, which could help them to achieve sustainable careers [19]. This generally means one’s talents and interests or the understanding of the opportunities and requirements of various career fields [20]. It ensures that youth have sufficient knowledge and understanding of their competencies, allowing them to select the occupational aspects of their future [21]. Wise et al. [22] presented a framework of career awareness that is related to three dimensions of work: the routines of work, the requisites of work, and the returns of work. Additionally, there are four significant aspects of careers: knowledge, values, preferences, and self-concepts.

Career awareness significantly affects career and talent development, so it is necessary to offer students continuous educational opportunities to enhance their career awareness [23]. Skills, knowledge, and attitudes are needed to improve career awareness [24], which can be applied through participating in career development courses. Ernst and Bowen [25] found that coursework can improve at-risk first-year engineering students’ career awareness. Korucu and Kabak [26] also pointed out that innovative interdisciplinary practices positively impact students’ career awareness through a meta-synthesis study. These show the possible effects of career development courses on career awareness. Thus, the following hypothesis is posited:

**Hypothesis** **1** **(H1):** *The more career-development-related courses that a student takes, the higher the student’s career awareness.*

### 2.2. Job Search Self-Efficacy

Derived from the self-efficacy defined by Bandura [27], job search self-efficacy is used to understand the initiation of and persistence in various coping behaviors [28]. Self-efficacy is related to career-relevant behavior [29], and assessing self-efficacy is a very useful strategy for testing whether it affects subsequent behavior in the career area [30]. Self-efficacy theory has been provided as an important model for the study of career development [31], which has been used to understand factors related to individuals’ degree of success in performing activities necessary for following and/or developing a career [32]. In 1983, Taylor and Betz [33] introduced self-efficacy to the career decision-making process by proposing a new concept called job search self-efficacy.

Many researchers have discussed the definition of job search self-efficacy. Solberg [34] thought that job search self-efficacy involved beliefs about one’s ability to perform career exploration activities successfully. Maddy III et al. [35] posited that job search self-efficacy is a specific efficacy that reflects one’s feelings about performing tasks that may lead to employment. Tolentino et al. [36] considered job search self-efficacy as the perceived general competence in seeking jobs. Teye-Kwadjo [37] built upon previous research findings and defined job search self-efficacy as the confidence in one’s ability to search for employment and gain employment successfully, and has long been found to be the most proximal determinant of employment among job seekers. In conclusion, job search self-efficacy is a psychological concept closely related to one’s career development, especially in job seeking.

It has been widely acknowledged and empirically supported that job search self-efficacy plays a central role in career development courses [38]. It is the responsibility of educational institutions and career providers to develop students’ job search self-efficacy. Furthermore, the development of job search self-efficacy programs applies to all individuals, including both high school students and college students [32]. One empirical study proved that the reception of social support is positively associated with job search self-efficacy and behavior [39]. Research findings have also illustrated the positive relationships between career development activities and job search self-efficacy. For example, Creed et al. [40] found that training significantly increases course participants’ job search self-efficacy. Lim et al. [41] determined that specific interventions, such as promising career coaching programs, facilitate job search behaviors. Cmar and McDonnall [42] utilized a quasi-experimental pre- and post-test to prove that job search skills training and summer work experience programs could increase participants’ job search self-efficacy. These studies implied that career development courses could influence university students’ job search self-efficacy. Thus, the following is hypothesized:

**Hypothesis** **2** **(H2):** *The more career-development-related courses that a student takes, the higher job search self-efficacy that the student will have.*

### 2.3. Career Planning Ability

Career planning ability is essential for developing one’s career path and acquiring occupational success. It is one competence of career development control [43], and it is closely related to the core concept of career maturity. It is demonstrated that a high career maturity equates to a strong planning ability [44]. Scholars from different countries have studied career planning abilities in different student groups, such as nursing students [45,46], vocational high school students [47], community college students [48,49], first-year students, and university students [1,50,51].

Career planning is generally regarded as choosing career goals and finding ways to achieve these goals [52]. In contrast, in existing studies, career planning ability is considered as the basis of the career planning process. Talib et al. [53] believed that career planning ability includes one’s personality and working environment, career information search, and career preparation. Young et al. [54] determined that career planning ability is an individual’s capability to plan a career trajectory in a given domain. Neureiter and Traut-Mattausch [55] posited that career planning ability is thinking about future career developments and actively formulating steps and plans to achieve one’s career goals. In China, career planning abilities usually consist of self-awareness, environment awareness, setting goals, making plans, and feedback correction [56].

There are three aspects influencing career planning ability: (1) knowledge and understanding of oneself, (2) knowledge and understanding of the working world, and (3) practical reasoning regarding the relationship between knowledge and self-understanding with the knowledge and understanding of the working world [52]. All of these are required in career development courses. A pre- and post-test of four-year undergraduate students showed that the faculty positively facilitates students’ career planning abilities through structured curricula [46]. Researchers in Malaysia found that career planning abilities could be improved if students follow a systematic career program [49]. Another two studies also supported that university students’ career planning ability could significantly increase through a career education module [51,53]. Therefore, it is reasonable to investigate the relationship between career development courses and career planning ability, as hypothesized below:

**Hypothesis** **3** **(H3):** *The more career-development-related courses that a student takes, the higher career planning ability that the student will have.*

## 3. Materials and Method

### 3.1. Data Sources and Procedure

The data for this study were gathered from a self-administrated survey: “The Effectiveness of College Students’ Career Development Courses” (ECSCDC). The survey items that measured students’ career awareness, job search self-efficacy, and career planning ability were derived from Crites and Savickas [57], Savickas and Porfeli [58], and Solberg et al. [31]. Partial but important items were selected and translated into Chinese to constitute the survey utilized for the present study. Using convenience sampling, faculty members at a large research university in an eastern province in China were recruited as survey distributors. They helped to share our survey link with students via a survey platform called WJX.CN while teaching undergraduate courses. Students were informed not to take the survey repeatedly if they had seen it before in their other classes. The students were notified that their personal information would be confidential and could not be identified through the study.

### 3.2. Participants

The survey collected undergraduate students’ individual characteristics, such as gender, ethnicity, and urbanicity, among others. It also contained students’ family characteristics, including annual income and parental occupations. Most importantly, it included the career-related information of students, such as career awareness, job search self-efficacy, career planning ability, and career course-taking, which were the primary variables of interest in this study.

To ensure that our sample was more representative, we adopted a similar approach to the one used in AI-Hanawi et al. [59], which used *Raosoft* website to calculate the needed sample size to represent the whole province’s undergraduate student’s population (approximately 115,000 undergraduates), with a 5% margin of error, 95% confidence level, and 50% response distribution. We obtained a recommended sample size of 385. In fact, after checking the validation of survey responses, 703 of the 708 responses were valid. After applying the listwise deletion method, no missing data were found in these 703 responses. As such, the final analytic sample of the present study consisted of these 703 responses. Among them, 62.87% were male students and 37.13% were female students. Concerning ethnicity, 93.88% were Han and 6.12% were from 55 other statutory minority groups, including Zhuang, Man, and Hui (see details in Table 1).

### 3.3. Measures

#### 3.3.1. Students’ Career-Related Outcomes

Students’ career-related outcomes in this study included three composited variables: students’ career awareness, job search self-efficacy, and career planning ability. These variables consisted of multiple survey items measured on a 5-point Likert scale, with 5 = Strongly Agree and 1 = Strongly Disagree. To examine whether the survey items could precisely measure the composited variables, we followed Zhao and Perez-Felkner [60] in order to conduct a reliability test, using Cronbach’s alpha and factor analysis using the principal-component factor method with orthogonal varimax rotation. Further details can be seen in Table 2.

***Career awareness***. The following questions were asked: “will you think about future jobs”, “are you aware of current choices”, “will you prepare for future jobs”, “are you aware of the importance of career planning”, “do you have career goal plans”, and “will you be aware of career development issues”. As shown in Table 2, Cronbach’s alpha for career awareness indicates a high reliability [61]. The factor loadings ranged from 0.76 to 0.84, meaning 57.76–70.56% of variances in these items could be explained by students’ career awareness.

***Job search self-efficacy***. Six questions were asked, including “will you actively gather all kinds of recruitment information”, “will you use social media to find job opportunities”, “will you use personal networking to find job opportunities”, “will you use campus activities to find job opportunities”, “are you familiar with resources to find satisfactory jobs”, and “are you familiar with satisfactory jobs”. These items also yielded a high reliability. In addition, the factor loadings were from 0.69 to 0.80, meaning 47.61–64.00% of the variances in these items could be explained by students’ job search self-efficacy.

***Career planning ability***. There were four items measuring students’ career planning ability: “will you be able to develop a practical career plan”, “will you be able to identify your career development goals”, “do you understand how to achieve your career goals”, and “do you know how to make a personal particular”. Table 2 displays a high Cronbach’s alpha, and the factor loadings were from 0.76 to 0.89, signifying that students’ career planning ability could explain 57.76–79.21% of the variances in these items.

#### 3.3.2. Career Course Taking

There were two primary predictors of students’ career course taking: whether or not a student has taken career courses (dichotomous variable) and the number of career courses that a student has taken (continuous variable). For the dichotomous variable, if a student has taken at least one career course, it was coded as “1”; otherwise, “0”. A total of 167 non-course participants and 536 course participants were collected. For the continuous variable, Table 1 presents that a student’s average number of career courses taken was 1.83.

#### 3.3.3. Demographic Characteristics

Whiston and Keller [62] comprehensively reviewed existing research focusing on the family’s influence on career development, finding that multiple factors, such as gender, race, and parents’ occupations, were likely to affect children’s career development. Based on this, other independent variables in our study include students’ demographic characteristics, such as gender (1 = men, 0 = women), ethnicity (1 = Han, 0 = Other ethnicity groups), urbanicity (where a student was originally from—urban, suburban, or rural), and college majors (whether science-related, liberal arts-related, or other majors). In addition, family characteristics, such as annual family income and parental occupations, were also considered. Following Zhao et al. [63], annual family income was sorted into low- (<CNY 30,000), middle- (>CNY 30,000 and <CNY 500,000), and high-income (>CNY 500,000) brackets. Furthermore, the mother’s and father’s occupations were, respectively, divided into four categories: government employee/military, service-related job, professional/expert, and other jobs. The detailed descriptive statistics can be viewed in Table 1.

### 3.4. Analytic Strategies

All of the analyses were performed in Stata 16. First, the descriptive statistics for the intended variables were provided. Then, by conducting a reliability test and factor analysis, we generated our three main outcome variables: career awareness, job search self-efficacy, and career planning ability. The analyses of the t-test and effect size (Cohen’s *d*) for a mean comparison were conducted to understand whether there are significant differences in career-related outcomes between female and male students [64]. Given that the new generated variables were continuous variables and the purpose of this study was to explore the intended relationships, three ordinary least squares (OLS) regression models were conducted to examine whether or not career courses affect students’ career awareness, job search self-efficacy, and career planning ability. The OLS can be explained as follows:

(1) $$Awareness=\beta_{0}+\beta_{1}*CD_{i}+\beta_{2}*Student_{i}+\beta_{3}*Family_{i},$$

(2) $$Job\;Search=\beta_{0}+\beta_{1}*CD_{i}+\beta_{2}*Student_{i}+\beta_{3}*Family_{i},$$

(3) $$Planning=\beta_{0}+\beta_{1}*CD_{i}+\beta_{2}*Student_{i}+\beta_{3}*Family_{i},$$

where *Awareness* in Equation (1) is students’ career awareness; *Job Search* in Equation (2) is students’ job search self-efficacy; and *Planning* in Equation (3) is students’ career planning ability. In Equations (1)–(3), $CD_{i}$ is whether or not a student has taken any career development courses; $Student_{i}$ is a vector of student *i*’s demographic characteristics, such as gender, ethnicity, urbanicity, and college majors; and $Family_{i}$ is a vector of student *i*’s family information, such as annual family income and parental occupations.

To understand to what extent career courses would affect these three outcomes, we further investigated these effects by replacing $CD_{i}$ with $NCD_{i}$—the number of career development courses a student has taken—which can be understood as follows:

(4) $$Awareness=\beta_{0}+\beta_{1}*NCD_{i}+\beta_{2}*Student_{i}+\beta_{3}*Family_{i},$$

(5) $$Job\;Search=\beta_{0}+\beta_{1}*NCD_{i}+\beta_{2}*Student_{i}+\beta_{3}*Family_{i}$$

(6) $$Planning=\beta_{0}+\beta_{1}*NCD_{i}+\beta_{2}*Student_{i}+\beta_{3}*Family_{i},$$

where *Awareness*, *Job Search*, *Planning*, $Student_{i}$, and $Family_{i}$ remained the same.

Before conducting these regression models, the variance inflation factor (VIF) was performed, where the largest VIF for mother’s occupations in others was less than 4, indicating that multicollinearity may not be an issue in this study.

Because we intended to use an identical set of independent variables to predict multiple continuous outcome variables, multiple analysis of variance (MANOVA) was performed [63] before conducting Equations (4)–(6) to test whether the number of career courses shows statistically significant variances in students’ career awareness, job search self-efficacy, and career planning ability, yielding a significant difference among the instances of career course taking in these three outcome variables, with Wilks’ Λ = 0.936, *F* (5, 697) = 3.13, and *p* < 0.001. In addition, other MANOVA indices, such as Pillai’s trace, Lawley–Hotelling trace, and Roy’s largest root, were all statistically significant at the 0.001 level.

After the regressions, post-estimation was performed to determine whether there were significant marginal effects, and was conducted using *margins* and *marginsplot* commands in Stata. In other words, we were interested in whether there were significant differences in students’ career awareness, job search self-efficacy, and career planning ability depending on the number of career courses taken. Since taking career courses was likely to have an additive effect on the relationships with the three outcome variables, a quadratic term of the number of career courses taken was added to visualize the intended relationships more clearly. Lastly, effect sizes (Cohen’s ƒ^2^) for linear regressions were also conducted for understanding the proportion of variability explained.

## 4. Results

### 4.1. Descriptive Results

Though some descriptive results were reported in the aforementioned *Measures* section, there are still some remaining descriptive statistics worth noting. Female students’ career awareness was slightly higher than their male peers, with *M* = 3.87, *SD* = 0.61 for women and *M* = 3.82, *SD* = 0.67 for men. In contrast, female students’ job search self-efficacy was slightly lower than their male peers, with *M* = 3.43, *SD* = 0.65 for women and *M* = 3.47, *SD* = 0.73 for men. For career planning ability, male students scored higher than female students. However, *t* tests indicated that none of these comparisons were statistically significantly different. In addition, Cohen’s *d* for career awareness, job search-self efficacy, and career planning ability was 0.074, −0.049, and −0.127, respectively. All of them were statistically insignificant and less than 0.2, indicating that the differences in career awareness, job search-self-efficacy, and career planning ability between male and female students were negligible [64]. Concerning students’ demographic characteristics, Table 1 shows that 45.38% of students were from rural areas, and 65.15% were majoring in science-related fields. The majority of students (68.84%) were from mid-income families. Other jobs comprised the largest share of father’s and mother’s occupations, with 31.15% and 41.39%, respectively. Looking at the skewness and kurtosis, the majority were in the acceptable ranges (−2 to 2 for skewness, and −7 to 7 for kurtosis) suggested by Byrne [65]. Though some were out of the ranges, Blanca et al. [66] pointed out that normality may not be the rule for the real data.

### 4.2. Career Development Courses and Students’ Career Awareness, Job Search Self-Efficacy, and Career Planning Ability

For all of the regression results, we reported unstandardized coefficients for an easier interpretation. Looking at Table 3, Model 1, Model 2, and Model 3 predicted students’ career awareness, job search self-efficacy, and career planning ability, respectively, from whether the students took at least one career course. Holding other variables constant, the career awareness and career planning ability of students who have taken a career course were, respectively, 0.096 (*p* < 0.05) and 0.147 (*p* < 0.001) higher than those of students who had not taken a career course. No significant association between job search self-efficacy and whether career courses were taken was observed.

In addition, college majors and annual family income seemed to be good predictors for students’ career awareness, job search self-efficacy, and career planning ability. For example, students from low-income families had a statistically significantly lower career awareness than those from high-income families, with *B* = −0.211, *p* < 0.05. Students majoring in liberal-arts-related disciplines were more likely to have a higher job search self-efficacy than those majoring in science-related majors, with *B* = 0.138, *p* < 0.05. Low-income students (*B* = −0.348, *p* < 0.01) and middle-income students (*B* = −0.279, *p* < 0.01) had lower career planning abilities than high-income students. Notably, male students were more likely to have a higher career planning ability than their female peers (*B* = 0.165, *p* < 0.05). Furthermore, effect sizes for Model 1–3 were 0.11, 0.11, and 0.17, respectively, indicating a medium effect [67].

### 4.3. Number of Career Development Courses Taken and Students’ Career Awareness, Job Search Self-Efficacy, and Career Planning Ability

**Figure 1:**
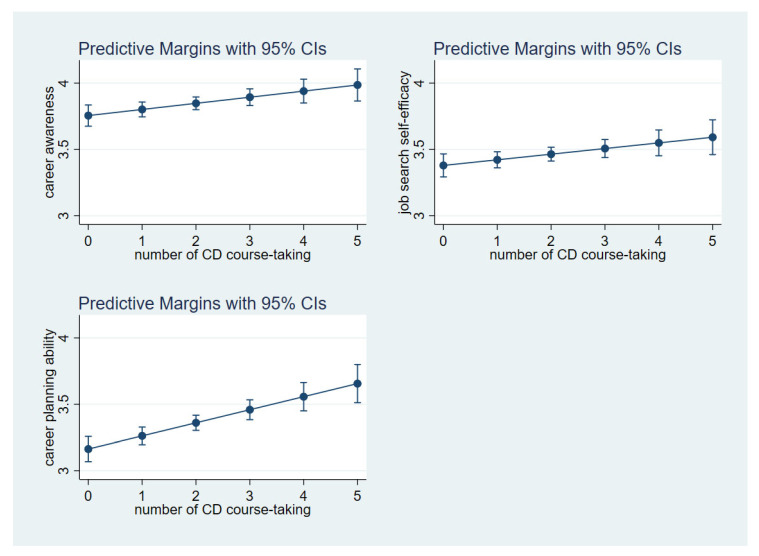
Predictive margins of career awareness, job search self-efficacy, and career planning ability by the number of career development courses taken.

Using OLS regressions, significant relationships were found between the number of career courses taken and students’ career awareness, job search self-efficacy, and career planning ability. Table 4 shows that, holding other variables constant, an additional career course was associated with a 0.099 unit increase in students’ career awareness. For job search self-efficacy and career planning ability, there was a 0.084 (*p* < 0.05) and 0.175 (*p* < 0.001) unit increase, respectively, if a student took an additional career course. Therefore, all three aforementioned hypotheses (H1, H2, and H3) were supported. Furthermore, gender, college majors, and annual family income were significantly associated with career awareness, job search self-efficacy, and career planning ability (see details in Table 4). Checking the effect sizes, Model 4–6 yielded a medium effect, with Cohen’s ƒ^2^ = 0.10, 0.10, and 0.15, respectively,

Focusing on students’ average career awareness, job search self-efficacy, and career planning ability at each point of career course taking (i.e., 0, 1, 2, 3, 4, 5), we found positive overall trends in Figure 1. This indicated that the more career courses that a student took, the higher the career awareness, job search self-efficacy, and career planning ability that the student had.

**Figure 2:**
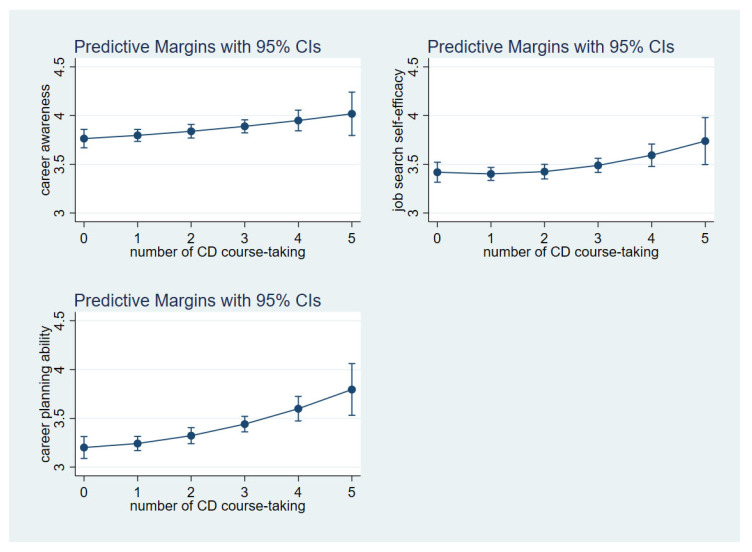
Predictive margins of career awareness, job search self-efficacy, and career planning ability with quadratic terms by the number of career development courses taken.

Figure 2 specifies the rates of changes in career awareness, job search self-efficacy, and career planning ability change. Visually, there is a slight increase in students’ job search self-efficacy with each career course taken for the lower level of job search self-efficacy. Each additional career course taken, starting from approximately three career courses, confirmed an increased job search self-efficacy. A similar pattern was found in the relationship between the number of career courses taken and students’ career planning ability.

## 5. Discussions

### 5.1. Effect of Career Development Courses on Students’ Career Awareness

Consistent with previous studies, the results of the present study show a positive relationship between career development courses and students’ career awareness [9,26]. In terms of career awareness, there is a significant difference between students who have taken career development courses and those who have not taken any career development courses. Students’ career awareness may sharply increase after taking only one career-development-related course, and the more courses that a student takes, the higher that student’s career awareness would be.

According to the career awareness framework [22], students’ career awareness includes four aspects: knowledge, values, preference, and self-concept. First, career development courses offer students the methodology used to gain needed information and the necessary tools to navigate today’s workforce. Second, the faculties of career development courses teach students what is essential when designing career paths and making occupational decisions. Third, in career development courses, students are inspired to think about their personalities, interests, abilities, and vision related to future work and life. Fourth, through lectures, discussions with peers, and different kinds of coursework, students can establish their own perceptions and opinions about their competence and goals. All of the above are seldom included in students’ professional courses but are centrally provided in career development courses. Therefore, students who have participated in career development courses have a greater opportunity to determine that they should think about and prepare for their careers compared to those who only take professional courses. In other words, career development courses help students to construct a higher degree of career awareness.

### 5.2. Effect of Career Development Courses on Students’ Job Search Self-Efficacy

Previous studies have shown that career development courses can facilitate job search self-efficacy [13,38,42,68]. This study finds it challenging to distinguish between the job search self-efficacy of students who have taken one career development course and those who have not taken any career development courses. However, this distinction is evident when students take more than two career development courses. The more courses that a student takes, the higher the job search self-efficacy that the student has. In other words, the adequate degree of a single career development course improves with the increase in the calculated number of courses that students have taken.

Bandura’s self-efficacy theory determines that self-efficacy can be acquired and modified through performance accomplishment, vicarious learning, verbal persuasion, and low levels of emotional arousal [69]. In career development courses, students are ordered to complete occupational development aptitude tests (such as the self-directed search), undergo mock interviews, practice career decision making, and participate in internships. Faculties and corporate mentors also encourage students to explore job opportunities and to help them establish confidence in order to acquire satisfying jobs. Thus, the more courses that a student takes, the more psychological facilitation and behavior guidance that the student will have, ultimately leading to a higher job search self-efficacy.

### 5.3. Effect of Career Development Courses on Students’ Career Planning Ability

This study investigated the relationship between career development courses and students’ career planning ability. The positive effect is reflected in two dimensions. Firstly, there is a significant improvement in students’ career planning ability after participating in career development courses, which has also been supported by previous studies [45,46,49,53]. Secondly, as an effect of career development courses on job search self-efficacy, our results reveal that the effect degree of career development courses deepened from the third course taken, demonstrating that the more courses that a student takes after taking two courses, the better the career planning ability that the student will have.

Career planning begins with self-knowledge. Students learn to set reasonable goals given basic information about themselves and their job environment, make a practical plan directed by career development course faculties, and adjust the program during their undergraduate studies and even while working [70]. It should be noted that all career planning-related abilities are lacking in students who are seldom in touch with the working world and mainly surround themselves with peers similar to them. Therefore, they need sufficient time to develop those abilities with guidance from course lecturers or those who have also taken career development courses. Different career development courses focus on certain objectives by concentrating on unique career planning ability aspects [71]. Teachers and classmates from different courses can bring various ideas into the fold. Therefore, with the increase in the number of courses taken, the student will think about their career more and will acquire more comprehensive abilities.

### 5.4. Practical Implications

According to statistics from the Ministry of Education, the number of higher education graduates in China is increasing every year and will break through 10 million in 2022 [72]. Therefore, students need a heightened career awareness, job search self-efficacy, and career planning ability to prepare for and react to this pressure. Previous studies [6,13,14] and our study jointly support the positive effect of career development courses on students’ career awareness, job search self-efficacy, and career planning abilities. Based on the findings, some practical solutions are suggested below.

First, universities could offer career development courses to all students and guarantee that every student can attend at least one class related to career development. Career planning courses are one of the most effective ways to improve students’ career development [73]. As the findings demonstrate, for students who have previously taken career courses, their career awareness and career planning ability were higher than those who had not taken any courses. As such, universities can provide a compulsory course related to career development. If it is listed as a graduation requirement, it would ensure that all students, regardless of their major, would enroll in one of these beneficial courses at some point during their undergraduate studies.

Second, universities could increase the number of career development courses offered and encourage students to take as many career development courses as possible. This is because the findings suggest that students experienced a higher improvement margin in their job search self-efficacy and career planning ability with each additional course taken after completing two courses. Universities could set a variety of optional career development courses for students. Therefore, relevant universities must invest more resources and teachers to construct these courses.

Third, this study finds that a student’s college majors, annual family income, and gender may influence that student’s career awareness, job search self-efficacy, and career planning ability. As such, universities should offer tailored career development courses to students with diverse backgrounds. For example, colleges can set particular career development courses based on students’ personal characteristics [74]. An institution such as a career development center could offer more course opportunities for students from low- and middle-income families and female career development courses for female students.

### 5.5. Limitations and Future Directions

One major limitation of the present study is that a causal relationship between career development courses and students’ career awareness, job search self-efficacy, and career planning ability cannot be concluded. To achieve causal inference, experiment designs or quasi-experimental designs such as propensity score matching and regression discontinuity are required in addition to specific data requirements. Future research could expand upon our study by collecting additional student information and counterfactually matching similar students and assigning them into a treatment group (those who have taken career development courses) and a control group (those who have not taken career development courses) by using propensity score matching to see the true effect of career development courses on students’ career-related outcomes. Another limitation is that our outcome variables are measured by self-administrated survey items, which may not precisely measure these psychological variables. However, it should be noted that the corresponding survey items are derived from the existing research [32,57,58]. We did not adopt all of the items, as this not only concerns barriers in terms of data collection, such as participants’ willingness to take the long survey, but also the conducting of reliability tests and factor analyses to enhance the measurement validity. Additionally, similar to how Zhao et al. [75] measured students’ psychological latent variables, structural regression models could be implemented to examine the relationships between career awareness, job search self-efficacy, and career planning ability. However, in some practical situations, it is still worth noting that career development courses help students to prepare for their future careers. As such, postsecondary administrators should create informed curriculum designs.

## 6. Conclusions

Understanding whether career development courses could potentially affect undergraduate students’ career-related outcomes is crucial. The results show that one career development course could significantly improve students’ career awareness and career planning ability. In addition, the present study also reveals a positive relationship between the number of career development courses taken with students’ career awareness, job search self-efficacy, and career planning ability, especially regarding the latter two. Furthermore, the findings demonstrate that a student’s major, annual family income, and gender could affect that individual’s career awareness, job search self-efficacy, and career planning ability. These findings provide the necessary foundation for postsecondary administrators to design courses that will ensure the students’ healthy and quality career development.

## Figures and Tables

**Table 1:** Descriptive statistics of students’ career course taking and demographic characteristics.

Variables	N	Mean	Skewness	Kurtosis
Career Development Courses				
CD Courses Taken	703	1.83	0.21	2.00
**Gender**				
Men	442	62.87%	−0.53	1.28
**Ethnicity**				
Han	660	93.88%	−3.66	14.41
**Urbanicity**				
Urban	299	42.53%	0.30	1.09
Suburban	85	12.09%	2.33	6.41
Rural	319	45.38%	0.19	1.03
**College Majors**				
Science	458	65.15%	−0.64	1.40
Liberal arts	183	26.03%	1.09	2.19
Other majors	62	8.82%	2.90	9.44
**Annual Family Income**				
High income	79	11.24%	2.45	7.03
Mid income	484	68.85%	−0.81	1.66
Low income	140	19.91%	1.51	3.27
**Father’s Job**				
Government/military	116	16.50%	1.80	4.26
Service-related	157	22.33%	1.33	2.77
Professional/expert	211	30.01%	0.87	1.76
Other job	219	31.15%	0.81	1.66
**Mother’s Job**				
Government/military	93	13.23%	2.17	5.71
Service-related	199	28.31%	0.96	1.93
Professional/expert	120	17.07%	1.75	4.06
Other job	291	41.39%	0.35	1.12

Note: Total N = 703.

**Table 2:** Factor analysis results of students’ career-related outcomes.

Variables	Cronbach’s α	Items	Factor Loadings	Means	SD
Career awareness	0.88	Think about future jobs	0.76	3.79	0.78
		Aware of current choices	0.76	3.96	0.82
		Preparedness for future jobs	0.84	3.88	0.81
		Aware of the importance of career planning	0.78	3.92	0.83
		Plans of career goals	0.82	3.64	0.85
		Aware of career development issues	0.80	3.85	0.81
Job search self-efficacy	0.84	Gather all kinds of recruitment information	0.78	3.37	0.97
		Use social media to find job opportunities	0.80	3.53	0.95
		Use personal networking to find job opportunities	0.69	3.50	0.97
		Use campus activities to find job opportunities	0.73	3.18	1.05
		Familiar with resources to find satisfactory jobs	0.78	3.55	0.85
		Familiar with your satisfactory jobs	0.73	3.60	0.82
Career planning ability	0.85	Develop a practical career plan	0.83	3.48	0.86
		Identify career development goals	0.88	3.38	0.95
		Understand how to achieve career goals	0.89	3.42	0.88
		Know how to make a personal particular	0.76	3.09	1.03

Note: N = 703. SD = standard deviation. All of the items were based on a 5-point Likert scale with 5 = Strongly Agree and 1 = Strongly Disagree.

**Table 3:** OLS regressions predicting students’ career awareness, job search self-efficacy, and career planning ability from career development course participants.

	Career Awareness (Model 1)		Job Search Self-Efficacy (Model 2)		Career Planning Ability (Model 3)	
	*B*	SE	*B*	SE	*B*	SE
Career Development Courses						
Whether takes CD courses	0.096 *	0.058	0.074	0.063	0.147 ***	0.069
**Gender**						
Men	0.002	0.058	0.105	0.063	0.165 *	0.069
**Ethnicity**						
Han	−0.005	0.103	0.065	0.112	−0.065	0.123
**Urbanicity**						
Suburban	−0.101	0.081	−0.023	0.088	−0.087	0.097
Rural	−0.074	0.058	−0.022	0.063	−0.058	0.069
**College Major**						
Liberal arts	0.060	0.065	0.138 *	0.070	0.109	0.078
Other majors	0.187 *	0.091	0.185	0.099	0.187	0.109
**Family Annual Income**						
Low income	−0.211 *	0.096	−0.192	0.104	−0.348 **	0.114
Mid income	−0.096	0.081	−0.201 *	0.088	−0.279 **	0.097
**Father’s Job**						
Service-related	0.047	0.088	−0.019	0.095	0.014	0.105
Professional/expert	0.026	0.090	0.019	0.098	−0.012	0.108
Other job	−0.026	0.093	−0.046	0.101	−0.065	0.111
**Mother’s Job**						
Service-related	−0.005	0.106	0.032	0.115	0.055	0.127
Professional/expert	−0.042	0.092	−0.077	0.100	−0.050	0.110
Other job	−0.046	0.093	−0.018	0.100	0.023	0.111
Constant	4.020 ***		3.534 ***		3.632 ***	
F test	1.72 *		1.29		2.58 ***	
Sample N	703		703		703	

Note: * *p* < 0.05, ** *p* < 0.01, *** *p* < 0.001. Women, other ethnicity group, urban, science major, high income, father’s government/military job, and mother’s government/military job are reference groups and are omitted from this table. SE = standard error. Standardized coefficients are reported for the variables of career development courses for model comparisons.

**Table 4:** OLS regressions predicting students’ career awareness, job search self-efficacy, and career planning ability from the number of career development courses taken.

	Career Awareness (Model 4)		Job Search Self-Efficacy (Model 5)		Career Planning Ability (Model 6)	
	*B*	SE	*B*	SE	*B*	SE
Career Development Courses						
CD courses taken	0.099 **	0.018	0.084 *	0.019	0.175 ***	0.021
**Gender**						
Men	0.009	0.058	0.111	0.063	0.178 **	0.069
**Ethnicity**						
Han	−0.017	0.103	0.054	0.112	−0.091	0.123
**Urbanicity**						
Suburban	−0.089	0.081	−0.012	0.088	−0.063	0.096
Rural	−0.069	0.058	−0.018	0.063	−0.051	0.069
**College Major**						
Liberal arts	0.059	0.065	0.136	0.070	0.102	0.077
Other majors	0.191 *	0.091	0.188	0.099	0.194	0.109
**Family Annual Income**						
Low income	−0.217 *	0.096	−0.197	0.104	−0.360 **	0.114
Mid income	−0.098	0.081	−0.202 *	0.088	−0.282 **	0.096
**Father’s Job**						
Service-related	0.049	0.088	−0.017	0.095	0.019	0.105
Professional/expert	0.031	0.090	0.026	0.098	0.005	0.107
Other job	−0.021	0.093	−0.041	0.101	−0.054	0.111
**Mother’s Job**						
Service-related	−0.014	0.106	0.023	0.115	0.033	0.126
Professional/expert	−0.044	0.092	−0.078	0.100	−0.054	0.110
Other job	−0.051	0.093	−0.023	0.100	0.013	0.110
Constant	3.907 ***		3.435 ***		3.404 ***	
F test	1.74 *		1.36		3.00 ***	
Sample N	703		703		703	

Note: * *p* < 0.05, ** *p* < 0.01, *** *p* < 0.001. Women, other ethnicity group, urban, science major, high income, father’s government/military job, and mother’s government/military job are reference groups and are omitted from this table. SE = standard error. Standardized coefficients are reported for the variables of career development courses for model comparisons.

## Data Availability

According to the data access policies, the data used to support the findings of this study are available from Zhejiang Academy of Higher Education, Hangzhou Dianzi University. Reasonable request for ECSCDC data is available through email: jingchao@hdu.edu.cn.

## References

[1] Bal E.A., Arikan S. (2020). The impact of a career development and planning course on university students’ career adaptability levels. Glob. Media J. Turk. Ed..

[2] Ciarocco N.J. (2018). Traditional and new approaches to career preparation through coursework. Teach. Psychol..

[3] Senter M.S. (2020). Implementing a careers and professional development course for sociology students. Teach. Sociol..

[4] Reese R.J., Miller C.D. (2010). Using outcome to improve a career development course: Closing the scientist-practitioner gap. J. Career Assess..

[5] Folsom B., Reardon R. (2003). College career courses: Design and accountability. J. Career Assess..

[6] Hansen J.M., Jackson A.P., Pedersen T.R. (2017). Career development courses and educational outcomes: Do career courses make a difference?. J. Career Dev..

[7] Australia M.M. (2010). Australian Blueprint for Career Development.

[8] Furbish D. (2012). An overview of new zealand career development services. Aust. J. Career Dev..

[9] Larkin J.E., Pines H.A., Bechtel K.M. (2002). Facilitating students’ career development in psychology courses: A portfolio project. Teach. Psychol..

[10] Henry P. (1993). Effectiveness of career-development courses for nontraditional premedical students: Improving professional identity. Psychol. Rep..

[11] Lucy C.A. (2017). Experiences and benefits of a career development course for undergraduate chemistry students. Anal. Bioanal. Chem..

[12] Troisi J.D. (2021). Improving student skills and career readiness through targeted and embedded instruction. Teach. Psychol..

[13] Reese R.J., Miller C.D. (2006). Effects of a university career development course on career decision-making self-efficacy. J. Career Assess..

[14] Ware M.E. (1981). Evaluating a career development course: A two year study. Teach. Psychol..

[15] Peng H. (2004). Taiwanese junior college students’ needs for and perceptions of a career planning course. Psychol. Rep..

[16] Jin L., Gao Y., Liu T., Creed P.A., Hood M. (2021). A comparison between flipped and lecture-based course delivery of a career development programme for chinese undergraduates. Br. J. Guid. Couns..

[17] Qiao Z., Jiang Y., Yang H., Guo X. (2013). The status quo, problems, and countermeasures of the career development and employment guidance courses in universities. China High. Educ. Res..

[18] Deng X., Zeng H., Liang M., Qiu J. (2022). Relations between different career-development profiles, academic self-efficacy and academic motivation in adolescents. Educ. Psychol..

[19] Wu J., Zhao T. (2022). Encouraging china’s college students to achieve sustainable careers: Evidence from structural equation modeling. Sustainability.

[20] Braverman M.t., Young J., King N., Paterson C., Weisskirch R. (2002). Career awareness and part-time work examined in lives of high school seniors. Calif. Agric..

[21] Dagyar M., Kasalak G., Ugurlu N. (2020). Academic career awareness and academic career interest among turkish undergraduate students. Cypriot J. Educ. Sci..

[22] Wise R., Charner I., Randour M.L. (1976). A conceptual framework for career awareness in career decision-making. Couns. Psychol..

[23] Hashish E.A.A. (2019). The effect of career awareness on perceived career and talent development self-efficacy and career barriers among nursing students. J. Res. Nurs..

[24] Johnson L.S. (2000). The relevance of school to career: A study in student awareness. J. Career Dev..

[25] Ernst J.V., Bowen B. (2014). Comparing career awareness opportunities of academically at-risk and non at-risk freshman engineering students. Am. J. Eng. Educ..

[26] Korucu A.T., Kabak K. (2021). The effects of stem and other innovative interdisciplinary practices on academic success, attitude, career awareness: A meta-synthesis study. J. Learn. Teach. Digit. Age.

[27] Bandura A. (1977). Self-efficacy: Toward a unifying theory of behavioral change. Psychol. Rev..

[28] Strauser D.R., Berven N.L. (2006). Construction and field testing of the job seeking self-efficacy scale. Rehabil. Couns. Bull..

[29] Lent R.W., Brown S.D., Larkin K.C. (1986). Self-efficacy in the prediction of academic performance and perceived career options. J. Couns. Psychol..

[30] Lent R.W., Hackett G. (1987). Career self-efficacy: Empirical status and future directions. J. Vocat. Behav..

[31] Solberg V.S., Good G.E., Nord D., Holm C., Hohner R., Zima N., Heffernan M., Malen A. (1994). Assessing career search expectations: Development and validation of the career search efficacy scale. J. Career Assess..

[32] Solberg V.S., Good G.E., Nord D. (1994). Career search self-efficacy: Ripe for applications and intervention programming. J. Career Dev..

[33] Taylor K.M., Betz N.E. (1983). Applications of self-efficacy theory to the understanding and treatment of career indecision. J. Vocat. Behav..

[34] Solberg V.S. (1998). Assessing career search self-efficacy: Construct evidence and developmental antecedents. J. Career Assess..

[35] Maddy III L.M., Cannon J.G., Lichtenberger E.J. (2015). The effects of social support on self-esteem, self-efficacy, and job search efficacy in the unemployed. J. Employ. Couns..

[36] Tolentino L.R., Sibunruang H., Garcia P.R.J.M. (2019). The role of self-monitoring and academic effort in students’ career adaptability and job search self-efficacy. J. Career Assess..

[37] Teye-Kwadjo E. (2021). The job-search self-efficacy (jsse) scale: An item response theory investigation. Int. J. Appl. Posit. Psychol..

[38] Lin Y.-J., Flores L.Y. (2013). Job search self-efficacy of east asian international graduate students. J. Career Dev..

[39] Russell J., Holmstrom A.J., Clare D.D. (2015). The differential impact of social support types in promoting new entrant job search self-efficacy and behavior. Commun. Res. Rep..

[40] Creed P.A., Bloxsome T.D., Johnston K. (2001). Self-esteem and self-efficacy outcomes for unemployed individuals attending occupational skills training programs. Community Work Fam..

[41] Lim D.H., Oh E., Ju B., Kim H.N. (2019). Mediating role of career coaching on job-search behavior of older generations. Int. J. Aging Hum. Dev..

[42] Cmar J.L., McDonnall M.C. (2021). Short-term effectiveness of job search skills training: Comparisons by summer work experience participation. Rehabil. Couns. Bull..

[43] Kuijpers M., Scheerens J. (2006). Career competencies for the modern career. J. Career Dev..

[44] Chen S., Zhou K. (2018). Career planning decision-making of college students based on cognitive science. NeuroQuantology.

[45] Donner G.J., Wheeler M.M. (2001). Career planning and development for nurses: The time has come. Int. Nurs. Rev..

[46] Waddell J., Spalding K., Canizares G., Navarro J., Connell M., Jancar S., Stinson J., Victor C. (2015). Integrating a career planning and development program into the baccalaureate nursing curriculum: Part i. Impact on students’ career resilience. Int. J. Nurs. Educ. Scholarsh..

[47] Hadi A., Aryani E., Suwidagdho D. (2020). The role of multiple intelligence on career planning of students in public vocational high school 3 klaten. KONSELI J. Bimbing. Dan Konseling.

[48] Talib J.A., Ariff A.M., Salleh A. (2010). The effects of career intervention program on community college students’ career development. Procedia-Soc. Behav. Sci..

[49] Bin Abu Talib J., Mohamad Z., Abdul Wahab N. (2015). Effects of career exploration module on career planning, career self-efficacy and career maturity among community college students. Mediterr. J. Soc. Sci..

[50] Jiang J. Research on the necessity of career planning education for freshmen. Proceedings of the 2018 International Workshop on Education Reform and Social Sciences (ERSS 2018).

[51] Teychenne M., Parker K., Teychenne D., Sahlqvist S., Macfarlane S., Costigan S. (2019). A pre-post evaluation of an online career planning module on university students’ career adaptability. J. Teach. Learn. Grad. Employab..

[52] Hariko R., Anggriana T.M. (2019). Reviewing the role of families in student career planning. Konselor.

[53] Talib J.A., Salleh A., Amat S., Ghavifekr S., Ariff A.M. (2015). Effect of career education module on career development of community college students. Int. J. Educ. Vocat. Guid..

[54] Young D.K., Carpenter D., Maasberg M. (2018). An examination of factors that influence students’ it career decisions. J. Comput. Inf. Syst..

[55] Neureiter M., Traut-Mattausch E. (2017). Two sides of the career resources coin: Career adaptability resources and the impostor phenomenon. J. Vocat. Behav..

[56] Gong K.G., GU X.Y. (2010). Development of undergraduates’ career planning competence questionnaire. Psychol. Explor..

[57] Crites J.O., Savickas M.L. (1996). Revision of the career maturity inventory. J. Career Assess..

[58] Savickas M.L., Porfeli E.J. (2012). Career adapt-abilities scale: Construction, reliability, and measurement equivalence across 13 countries. J. Vocat. Behav..

[59] Al-Hanawi M.K., Angawi K., Alshareef N., Qattan A.M.N., Helmy H.Z., Abudawood Y., Alqurashi M., Kattan W.M., Kadasah N.A., Chirwa G.C. (2020). Knowledge, attitude and practice toward COVID-19 among the public in the kingdom of saudi arabia: A cross-sectional study. Front. Public Health.

[60] Zhao T., Perez-Felkner L. (2022). Perceived abilities or academic interests? Longitudinal high school science and mathematics effects on postsecondary stem outcomes by gender and race. Int. J. STEM Educ..

[61] Kline P. (2013). Handbook of Psychological Testing.

[62] Whiston S.C., Keller B.K. (2004). The influences of the family of origin on career development: A review and analysis. Couns. Psychol..

[63] Zhao T., Su Q., Hu X. (2022). The relationships between family characteristics and undergraduate students’ COVID-19 responses: A cross-sectional study in china. Front. Public Health.

[64] Sullivan G.M., Feinn R. (2012). Using effect size-or why the p value is not enough. J. Grad. Med. Educ..

[65] Byrne B.M. (2013). Structural Equation Modeling with Mplus: Basic Concepts, Applications, and Programming.

[66] Blanca M.J., Arnau J., López-Montiel D., Bono R., Bendayan R. (2013). Skewness and kurtosis in real data samples. Methodol. Eur. J. Res. Methods Behav. Soc. Sci..

[67] Cohen J. (2013). Statistical Power Analysis for the Behavioral Sciences.

[68] Muceldili B., Artar M., Erdil O. (2021). Determining the moderating role of career guidance in the relationship between social isolation and job search self-efficacy: A study on university students. Bus. Econ. Res. J..

[69] Heffernan C.J. (1988). Social foundations of thought and action: A social cognitive theory, albert bandura englewood cliffs, New Jersey: Prentice hall, 1986, xiii+ 617 pp. Hardback. Us $39.50. Behav. Chang..

[70] Greenhaus J.H., Kopelman R.E. (1981). Conflict between work and nonwork roles: Implications for the career planning process. Hum. Resour. Plan..

[71] Cheung R., Jin Q. (2016). Impact of a career exploration course on career decision making, adaptability, and relational support in hong kong. J. Career Assess..

[72] The Class of 2022 College Graduates Will Break 10 Million for the First Time. http://www.moe.gov.cn/jyb_xwfb/s5147/202111/t20211122_581508.html.

[73] Grier-Reed T., Chahla R. (2015). Impact of a constructivist career course on academic performance and graduation outcomes. J. Coll. Stud. Retent. Res. Theory Pract..

[74] Bennett D., Knight E., Bell K. (2020). Graduate employability and the career thinking of university stemm students. Teach. High. Educ..

[75] Zhao T., Zhang Y., Wu C., Su Q. (2021). Will anti-epidemic campus signals affect college students’ preparedness in the post-covid-19 era?. Int. J. Env. Res. Public Health.

